# Supplementary material for: Metabolites from Aerial Parts of Glycyrrhiza foetida as Modulators of Targets Related to Metabolic Syndrome
Source: Biomolecules. 2024 Apr 11;14(4):467. doi: 10.3390/biom14040467 (PMC11048066; doi:10.3390/biom14040467)
Supplement: Supplementary file 1 [file biomolecules-14-00467-s001.zip › biomolecules-2912290-supplementary.pdf]

Supporting data

Metabolites from aerial parts of *Glycyrrhiza foetida* as modulators of targets related to metabolic syndrome

Hekmat B. Al-Hmadi[1,2], Elena Serino[3], Arianna Pastore[3], Giuseppina Chianese[3], Saoussen Hammami[2], Mariano Stornaiuolo[3,*] and Orazio Taglialatela-Scafati[3,*]

[1] Department of Chemistry, College of Medicine, AL-Muthanna University, Samawah, Iraq; hekmat.alhmadi@mu.edu.iq (H.A-H.)

[2] Laboratory of Environmental Chemistry and Clean Processes (LR21ES04), Faculty of Sciences of Monastir, Monastir University, Monastir, 5000, Tunisia; h_saoussen@yahoo.fr (S.H.)

[3] Department of Pharmacy, School of Medicine and Surgery, University of Naples Federico II, Via D. Montesano 49, 80131 Napoli, Italy; elena.serino@unina.it (E.S.); g.chianese@unina.it (G.C.); arianna.pastore@unina.it (A.P.)

Figure S1: ^{1}H NMR spectrum of compound **5** ($CDCl_3$, 600MHz) 2

Figure S2: HSQC 2D NMR spectrum of compound **5** ($CDCl_3$, 600MHz) 2

Figure S3: HMBC 2D NMR spectrum of compound **5** ($CDCl_3$, 600MHz) 3

Figure S4: COSY 2D NMR spectrum of compound **5** ($CDCl_3$, 600MHz) 3

Figure S5: ^{1}H NMR spectrum of compound **21** ($CDCl_3$, 600MHz) 4

Figure S6: HSQC 2D NMR spectrum of compound **21** ($CDCl_3$, 600MHz) 4

Figure S7: HMBC 2D NMR spectrum of compound **21** ($CDCl_3$, 600MHz) 5

Figure S8: COSY 2D NMR spectrum of compound **21** ($CDCl_3$, 600MHz) 5

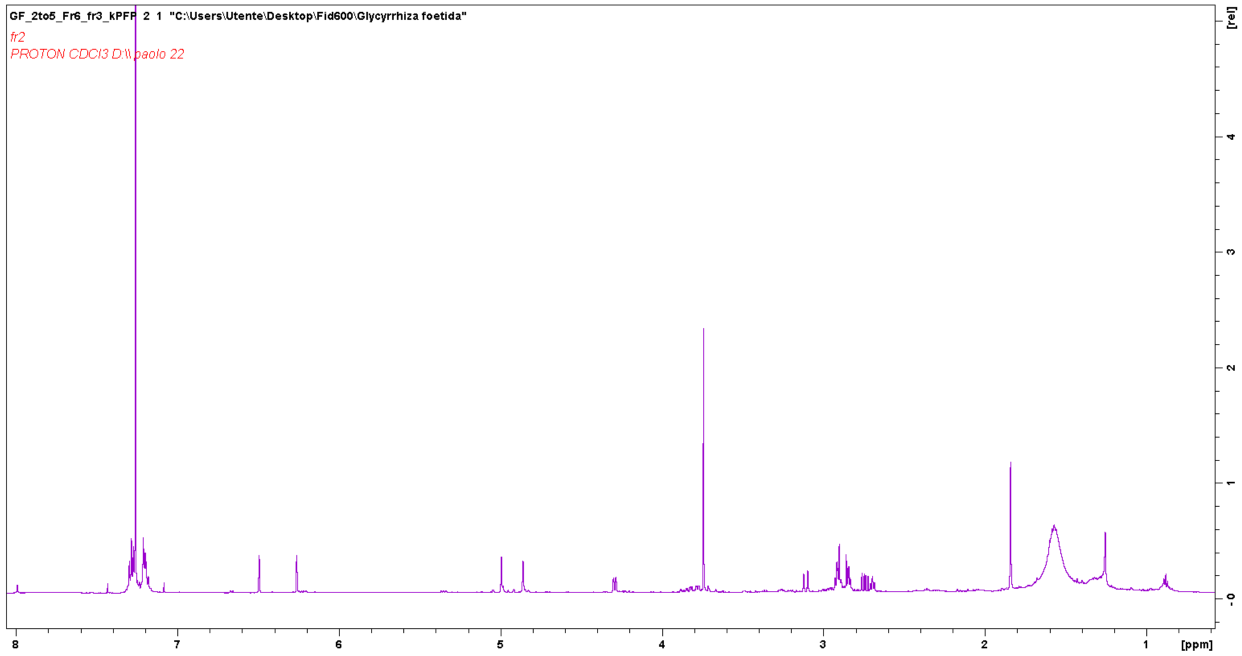

Figure S1: ^{1}H NMR spectrum of compound **5** ($CDCl_3$, 600MHz).

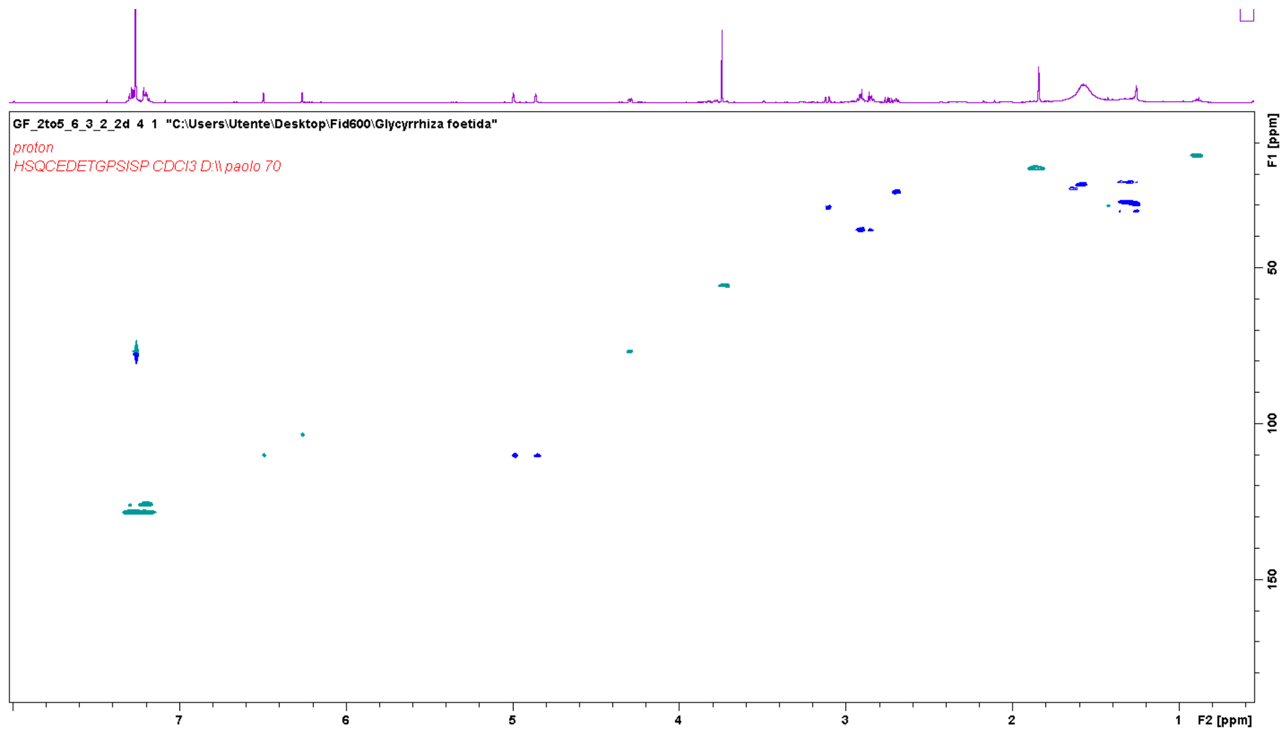

Figure S2: HSQC 2D NMR spectrum of compound **5** ($CDCl_3$, 600MHz).

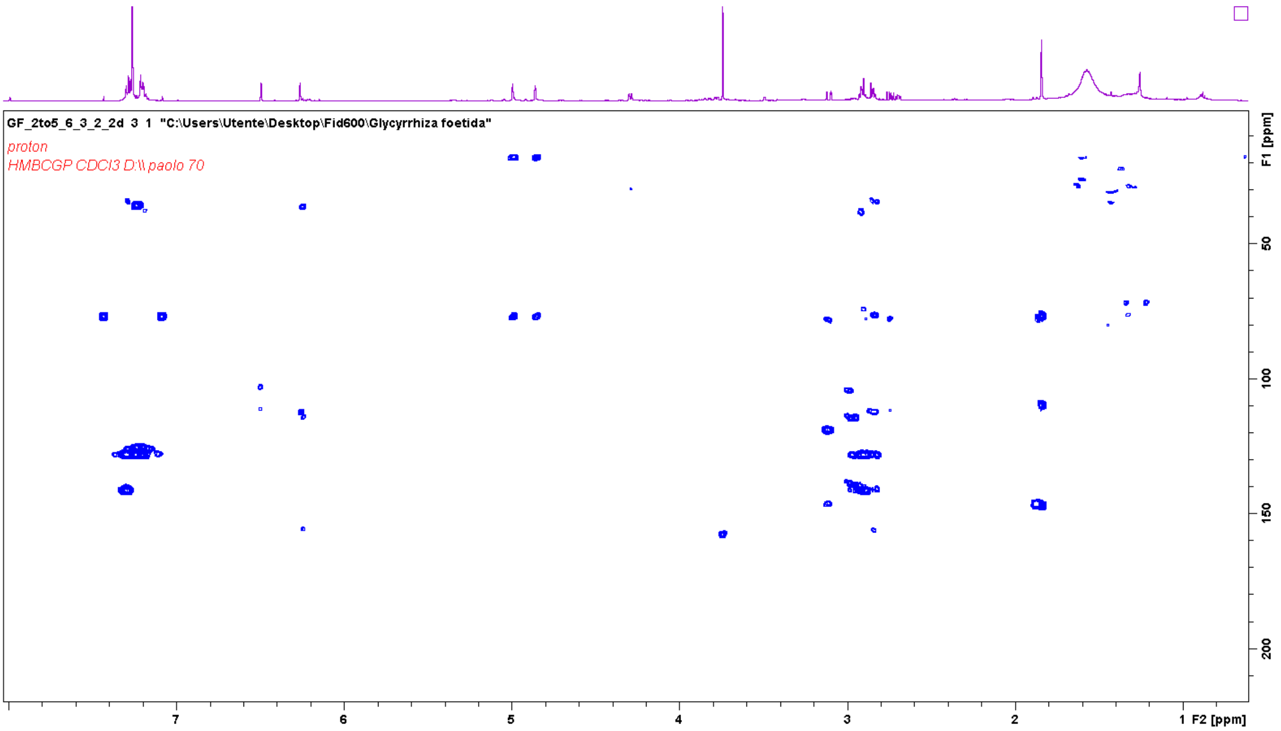

Figure S3: HMBC 2D NMR spectrum of compound **5** ($CDCl_3$, 600MHz).

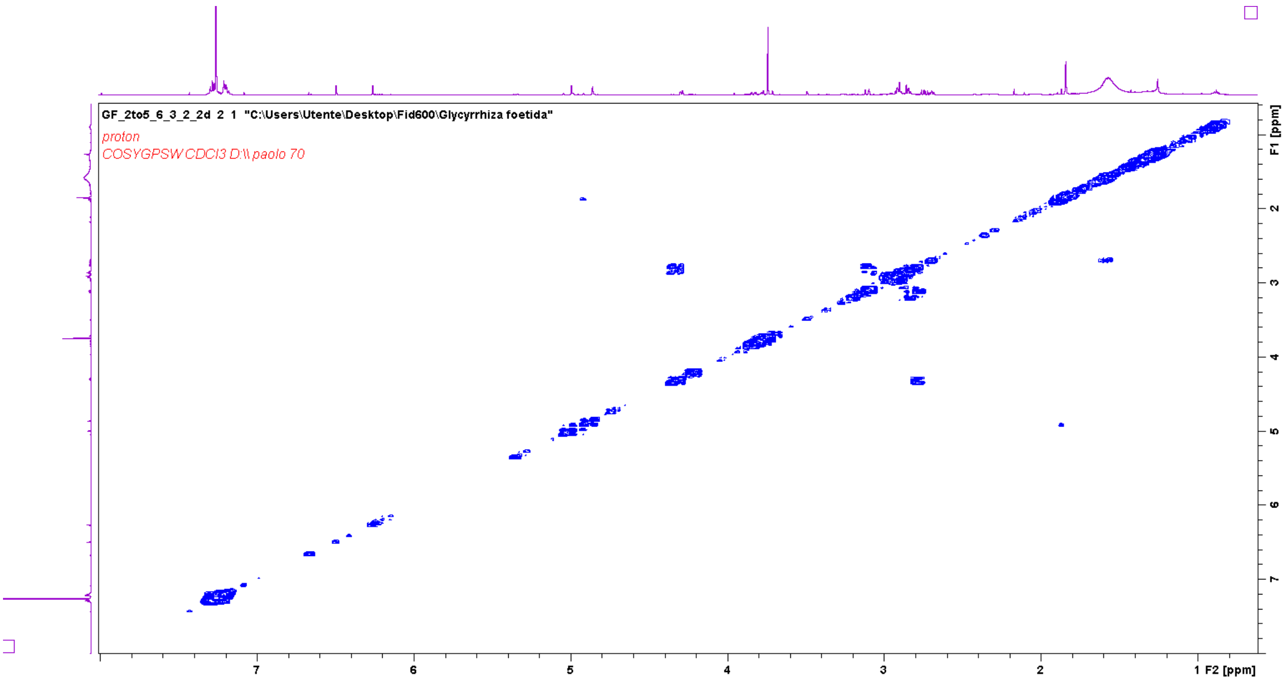

Figure S4: COSY 2D NMR spectrum of compound **5** ($CDCl_3$, 600MHz).

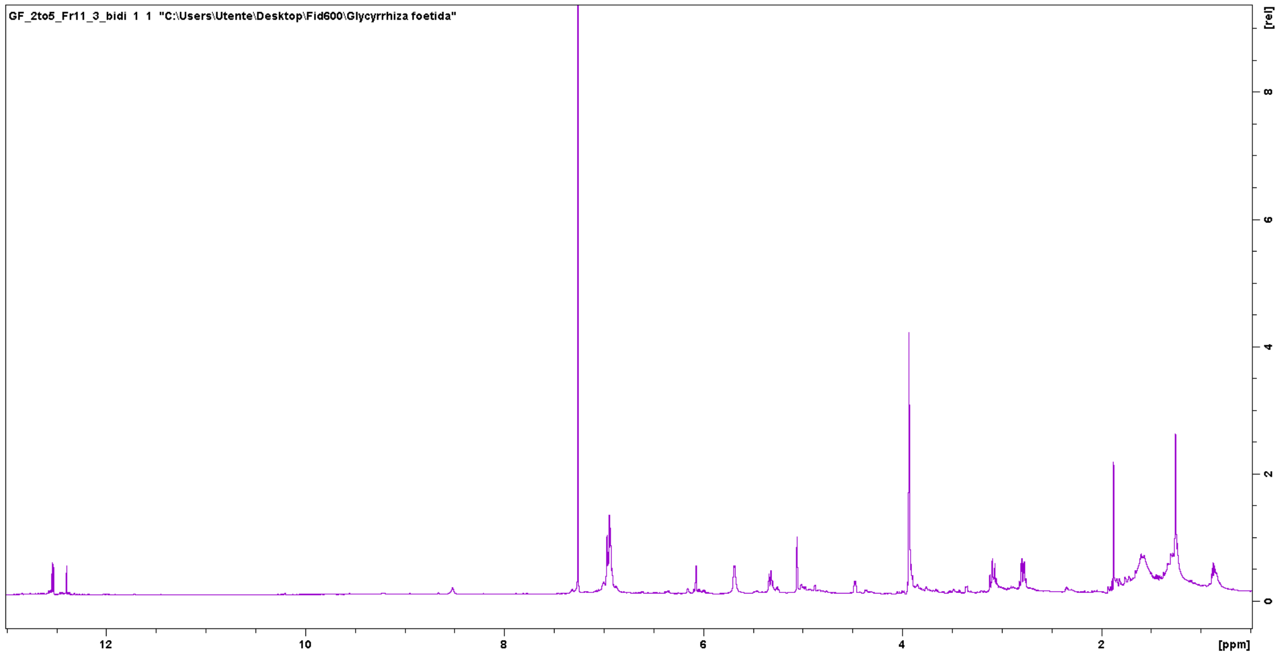

Figure S5: ^{1}H NMR spectrum of compound **21** (CDCl$_3$, 600MHz).

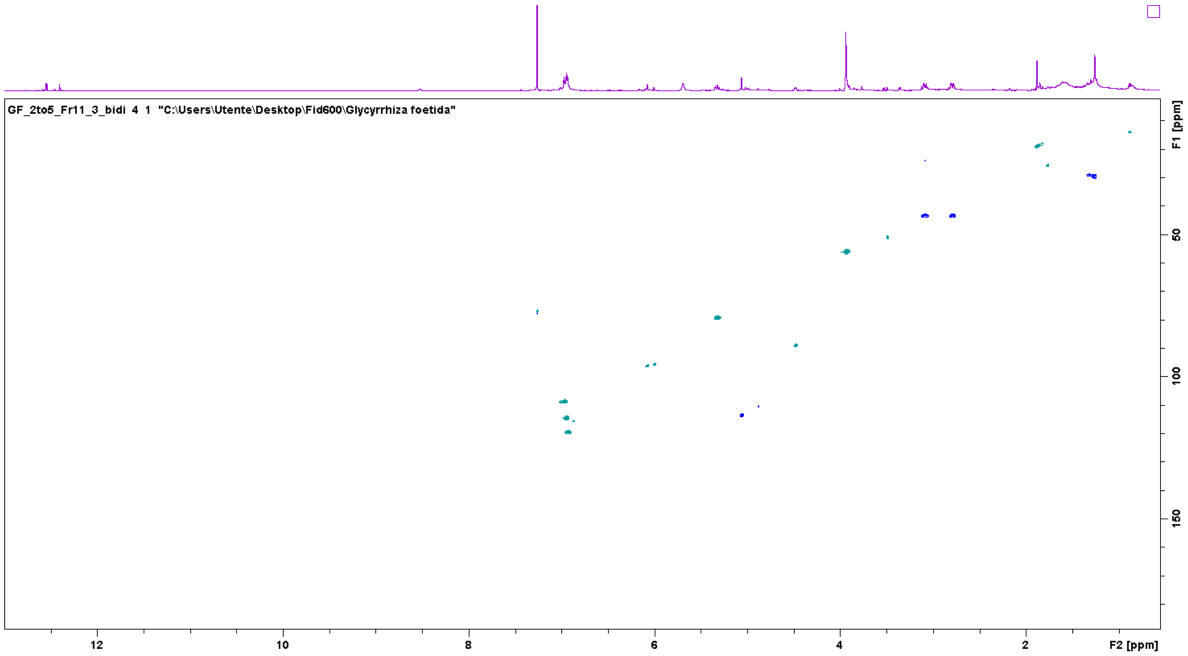

Figure S6: HSQC 2D NMR spectrum of compound **21** (CDCl$_3$, 600MHz).

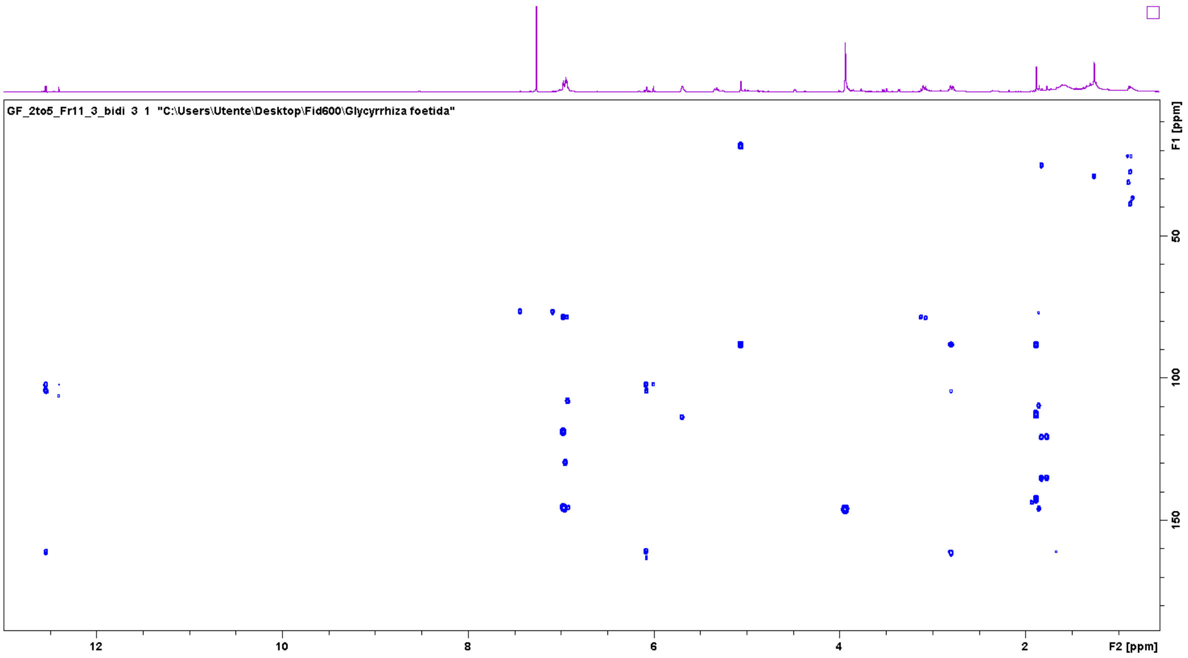

Figure S7: HMBC 2D NMR spectrum of compound **21** ($CDCl_3$, 600MHz).

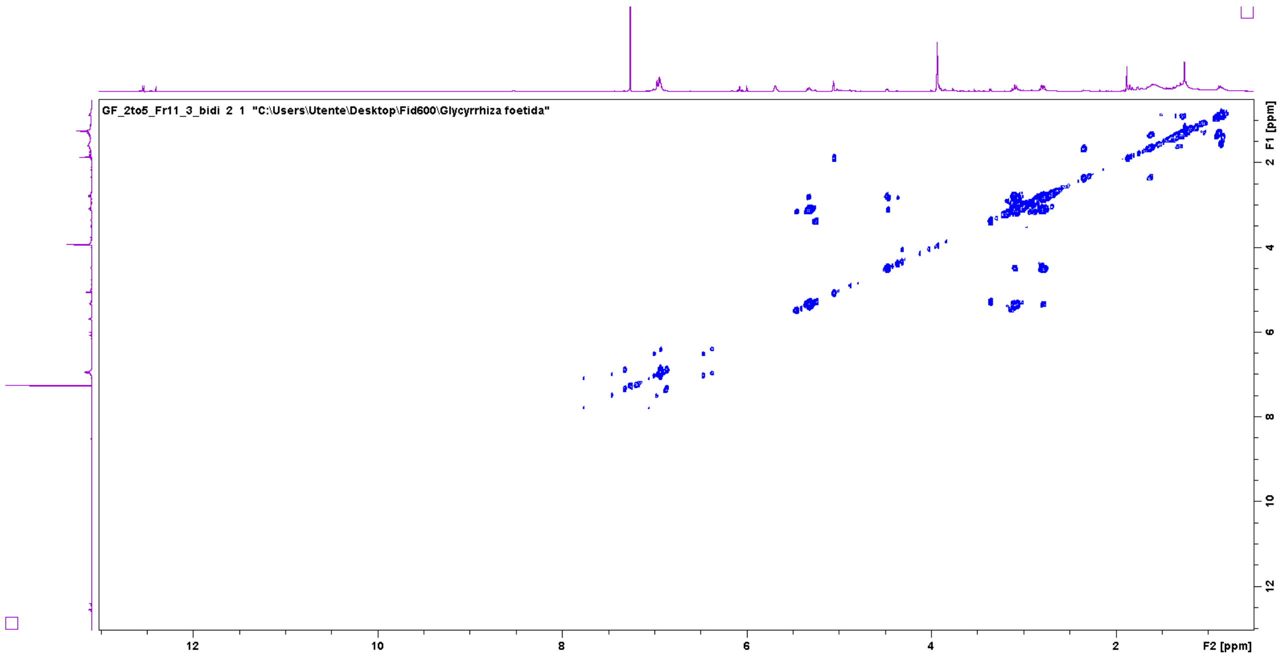

Figure S8: COSY 2D NMR spectrum of compound **21** ($CDCl_3$, 600MHz).
